# Giant phonon anomaly associated with superconducting fluctuations in the pseudogap phase of cuprates

**DOI:** 10.1038/ncomms10378

**Published:** 2016-01-20

**Authors:** Ye-Hua Liu, Robert M. Konik, T. M. Rice, Fu-Chun Zhang

**Affiliations:** 1 Theoretische Physik, ETH Zurich, 8093 Zurich, Switzerland; 2 Condensed Matter Physics and Material Science Department, Brookhaven National Laboratory, Upton, New York 11973, USA; 3 Department of Physics, Zhejiang University, Hangzhou 310027, China; 4 Collaborative Innovation Center of Advanced Microstructures, Nanjing 210093, China

## Abstract

The pseudogap in underdoped cuprates leads to significant changes in the electronic structure, and was later found to be accompanied by anomalous fluctuations of superconductivity and certain lattice phonons. Here we propose that the Fermi surface breakup due to the pseudogap, leads to a breakup of the pairing order into two weakly coupled sub-band amplitudes, and a concomitant low energy Leggett mode due to phase fluctuations between them. This increases the temperature range of superconducting fluctuations containing an overdamped Leggett mode. In this range inter-sub-band phonons show strong damping due to resonant scattering into an intermediate state with a pair of overdamped Leggett modes. In the ordered state, the Leggett mode develops a finite energy, changing the anomalous phonon damping into an anomaly in the dispersion. This proposal explains the intrinsic connection between the anomalous pseudogap phase, enhanced superconducting fluctuations and giant anomalies in the phonon spectra.

## Abstract (web summary)

The emergence of a giant phonon anomaly in the pseudogap phase of underdoped cuprate superconductors has been assumed to be a consequence of instability towards a charge density wave state. Here, the authors present a theory suggesting the anomaly arises due to large superconducting fluctuations.

## Graphical abstract

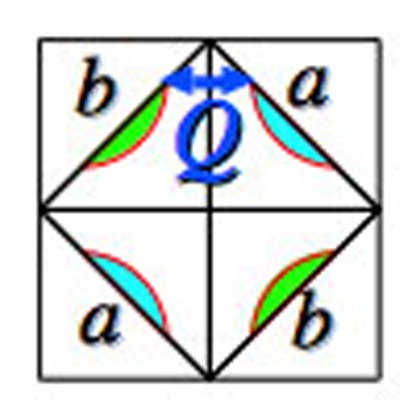

The unexpected discovery of a giant phonon anomaly (GPA) in the dispersion of low energy phonons in underdoped pseudogap cuprates has stimulated reconsideration of the role of phonons in high-critical temperature (high-*T*_c_) cuprate superconductors^1^,^2^,^3^,^4^,^5^. Recently many groups have proposed these anomalies are caused by other electronic instabilities, for example, charge density wave (CDW) order and also pair density wave order, which compete with the uniform *d*-wave pairing state^6^,^7^,^8^,^9^,^10^,^11^,^12^,^13^. A novel proposal has been put forward by Lee, who argues that Amperean pairing occurs in the pseudogap phase leading to an instability towards pair density wave and also CDW order^14^.

Lattice fluctuations associated with the GPA have a dynamic nature, as argued by Le Tacon *et al.*^4^,^15^, while random static CDW can still be induced by local perturbations, such as the random acceptors in nearly all underdoped cuprates^16^. Recent NMR/nuclear quadrupole resonance experiments on yttrium barium copper oxides found evidence for static lattice distortions, possibly induced around lattice imperfections by GPA. However, systematic splitting of the NMR lines, which would be the evidence for long-range ordered CDW^16^, has not been observed. Even for the cleanest stoichiometric underdoped cuprate YBa_2_Cu_4_O_8_, experiments by Suter *et al.*^17^ found only dynamic charge fluctuations but no static lattice ordered modulation, in agreement with earlier NMR experiments^18^,^19^.

Two recent studies of yttrium barium copper oxide samples covering a range of hole densities *p*, found an onset hole density *p*_c1_∼0.18 for the lattice anomalies, which coincides with the onset of the pseudogap^15^,^16^. Early angle-resolved photoemission spectroscopy (ARPES) experiments found that the onset of the pseudogap is characterized by a breakup of the Fermi surface into four pieces centred on the nodal directions^20^. A rapid expansion of the temperature range of superconducting (SC) fluctuations above the transition temperature for long-range superconductivity, *T*_c_ (*p*), is also observed^21^.

The unique combination of the onset of the GPA in hole doping, coinciding with Fermi surface breakup, and the onset of the GPA in temperature, coinciding with the onset of SC fluctuations, leads us to examine possible consequences of the special disconnected nature of the Fermi surface in the pseudogap phase, on *d*-wave superconductivity. We find that SC fluctuations in an extended temperature above *T*_c_ can result as a special feature of *d*-wave superconductivity in the presence of a disconnected Fermi surface in the pseudogap phase. We shall show below how these enhanced SC fluctuations in turn can couple to finite wavevector phonons leading to GPA.

## Results

### Electronic structure of the pseudogap phase

**Figure 1:**
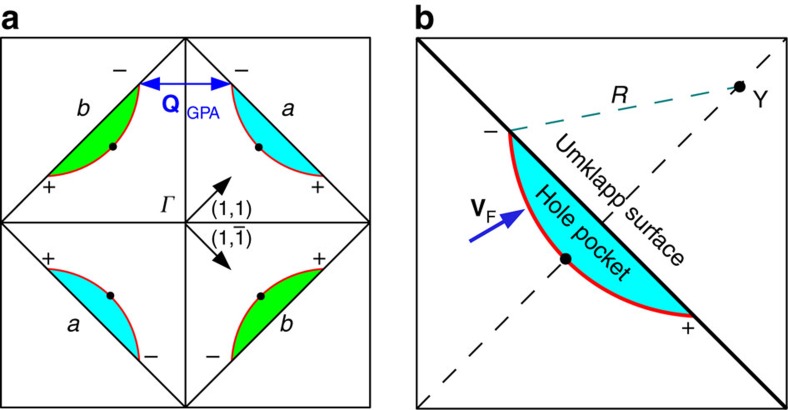
Representation of the band structure by Fermi arcs. (**a**) The breakup of Fermi surface to 2 sub-bands *a* and *b* in (1, 1) and 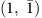 directions. **Q**_GPA_ is a wavevector connecting the two sub-bands, which is also the wavevector of the phonon anomaly. (**b**) Simplified model of Fermi arcs. Each Fermi arc is represented by a circular arc (shown red) with center **Y**, radius *R*, and terminates at the Umklapp surface. The Fermi velocity **v**_F_ (blue arrow) is assumed to a have constant magnitude on the whole arc. **Y** is uniquely determined by the choices of the wavevector between arc tips to be 0.51*π*, and the hole concentration *p*=11.5%. These are typical values in ARPES experiments. ± are the signs of the *d*-wave symmetry factor *γ*_**p**_ at different regions in the Brillouin zone. Black dots on the arcs indicate positions of superconducting nodes.

To describe the electronic state in the pseudogap phase, we use the Yang–Rice–Zhang model, which was put forward by two of us some years ago^22^,^23^. In this model the single-particle propagator was chosen to have a *d*-wave pairing self-energy, but with the crucial difference that the pairing gap opens up not on the Fermi surface, but on a special surface in the momentum space (or **k**-space), which was called the Umklapp surface (U-surface). This is a square surface connecting the antinodal points (±*π*, 0) and (0, ±*π*) in the Brillouin zone, as shown in Fig. 1. The underlying idea is to generalize the conditions that give rise to the D-Mott insulating phase in the one-dimensional (1D) case of the exactly half-filled two-leg Hubbard ladder, to the case of a square planar Hubbard model close to half filling. The D-Mott phase in 1D occurs already at weak coupling, which allows a complete analysis by a combination of one-loop renormalization group and bosonization methods^24^,^25^. The special feature of the D-Mott insulator is that it has an isolated groundstate with finite energy gaps in both charge and spin sectors. It follows that both the *d*-wave pairing correlations and commensurate antiferromagnetic correlations are strictly short ranged. Hence, the term Mott insulator can be applied to this insulating state with strictly short range correlations driven by the onsite Coulomb interactions. The origin of this behaviour can be traced back to the presence of several Umklapp processes (U-processes) which span the 4 Fermi points of the ladder Fermi surface exactly at half filling. These U-processes turn the metallic state with a Fermi volume of 4*π*, into an insulator with strictly short range correlations. The existence of finite gaps in both the one-particle and two-particle spectra and in the spin spectrum are special features of this state.

Early one-loop renormalization group calculations by Honerkamp *et al.*^26^,^27^ on the square lattice Hubbard model with both nearest neighbour and next-nearest neighbour hopping terms found strong *d*-wave pairing and antiferromagnetic correlations appearing at low hole densities as the magnitude of the onsite Coulomb interaction is increased. This behaviour is analogous to the case of the D-Mott insulator discussed above. Further it suggests a special role for the U-surface. Note each **k**-point on this surface belongs by symmetry to a set of 8 points, which are spanned by additional U-processes analogous to the D-Mott case with Fermi points in 1D. Note these 8 **k**-points are degenerate in energy by square symmetry, although the U-surface itself is not a constant energy surface in the presence of next-nearest neighbour hoppings.

The U-surface encloses an area of exactly one-electron per site. If we start from an excited single-electron state with finite hole doping, which has all occupied states inside the U-surface, there will be an empty nodal Fermi arc inside the U surface remaining. Each arc encloses an area of a quarter of the total hole concentration (see Fig. 1). These four Fermi arcs are not spanned by U-processes so that a *d*-wave SC gap can open along these arcs in a SC state. Later detailed ARPES experiments examined the predictions of the Yang–Rice–Zhang model carefully and confirmed that arcs actually are anisotropic pockets with spectral weight concentrated on the inner surfaces that are closest to the zone center^28^.

Turning to the SC state, the SC complex pairing amplitude is confined to two disconnected pairs of pockets centred on the nodal in the (1, 1) and 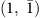 directions, as illustrated in Fig. 1. We shall refer to these two sets of Cooper pairs as sub-band *a* and *b*, respectively. An examination of the pair scattering processes shows that there are substantial intra-sub-band (*π*, *π*) scattering processes, which should act to stabilize the nodes lying along the diagonal directions (1, 1) and 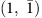. Assuming that the groundstate retains *d*-wave pairing symmetry, we arrive at a phase distribution illustrated in Fig. 1a. The breakup of the superconductivity into *a* and *b* sub-bands opens the possibility of SC phase fluctuations not just of the overall Josephson phase, but also of the phase difference between the two disconnected sets of Cooper pairs. The possibility of such phase fluctuations between separated pieces of the Fermi surface in multiband *s*-wave superconductors, was studied by Leggett many years ago^29^. He showed that when inter-band Cooper pair scattering is weak compared to intra-band scattering, a new low energy collective Leggett mode (LM) appears.

The inter-sub-band processes that transfer Cooper pairs between sub-bands *a* and *b* involve competition between same and opposite sign pairing amplitudes with only slight differences in the single-quasiparticle momentum transfers. Note to favour pairing between same phase regions the effective interaction should be attractive while repulsive interactions give depairing contributions. Scattering processes between opposite sign phase regions obey the opposite rules, that is, effective attractive interactions are depairing but repulsive ones are pairing. Note all these scattering processes involve similar wavevectors in the present case of a *d*-wave state. For this reason when we separate the pairing scattering in terms of intra-sub-band and inter-sub-band processes for a *d*-wave state, we see it is plausible to propose that the intra-sub-band processes are dominant relative to only weak inter-sub-band processes. This suggests the existence of a low-lying LM and leads us to investigate its influences to the exchange of Cooper pairs between the *a* and *b* sub-bands.

The presence of two distinct SC order parameters, 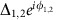, one for each pair of diagonal pockets, will lead to a wide region in temperature above *T*_c_ of enhanced SC fluctuations. Because of the Josephson coupling between *φ*_1_ and *φ*_2_, vortices in the phase difference *φ*_1_−*φ*_2_ will be suppressed, following the arguments given in ref. 30. This suppression of vortices antisymmetric in the phase will in turn reduce the fugacity of vortices symmetric in the phases (that is, *φ*_1_+*φ*_2_), leading to an extended temperature range where, while vortices are unbound, their density is less than it would be absent the inter-phase Josephson coupling. We will consider a detailed analysis of this phenomenon in future work. We do note, however, that this framework provides a natural means to understand the extended temperature range in which a *c* axis intra-bilayer Josephson plasmon is found to exist in optical conductivity experiments on YBa_2_Cu_3_O_7−*δ*_ (ref. 21).

Last for simplicity the anisotropic nodal Fermi pockets will be represented as simply as Fermi arcs (Fig. 1) with a constant Fermi velocity *v*_F_ along the arc. The parameters are chosen from a recent paper by Comin *et al.*^31^

### Leggett mode and fluctuations

Assuming that both intra- and inter-sub-band couplings *U* and *J* are separable *d*-wave forms with symmetry factor, *γ*_**p**_, allows us to obtain the fluctuation pair propagator from the following Bethe–Salpeter equation (Supplementary Fig. 1)


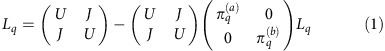


for *L*_*q*_, in terms of which the full anisotropic fluctuation is written as *L*_*pp*′*q*_=*γ*_**p**_*L*_*q*_*γ*_**p**′_. Here *p*, *p*′ and *q* denote both momentum and frequency, for example, *q*=(**q**, *iq*_0_). The electronic bubble 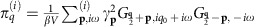 is defined on both sets of Fermi arcs for *i*=*a*, *b*. We denote zero temperature, finite temperature and retarded Green's functions by *G*_**p**,*ω*_, *G*_**p**,*iω*_, and 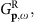 respectively, and similarly for other quantities. In the temperature region *T*_c_<*T*<*T*_o_ (with *T*_o_ the onset temperature of SC fluctuations), the electron's retarded Green's function takes the form 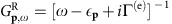, where 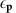 is the Fermi arc dispersion and Γ^(e)^=*aT*+*bT*^2^ is a temperature dependent quasiparticle damping^32^. The retarded propagator for the fluctuating LM is derived to be


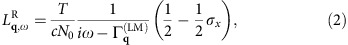


where *σ*_*x*_ is the Pauli matrix. This form of the fluctuation propagator describes an overdamped bosonic mode, see Supplementary Note 1 for details. Here *N*_0_ is the density of states per spin at the Fermi energy for one pair of arcs, the constant 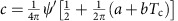 with 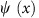 the digamma function. The damping of the LM 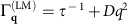, in which the inverse relaxation time 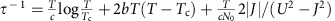 and the diffusion constant 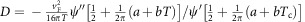 are functions of the temperature. The uniform damping rate 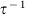 decreases as long-range order at *T*_c_ is approached.

For the ordered phase, we take zero temperature as a representative. In this case the LM is derived similarly to equation (1), but with a different form of the electronic bubble^29^,^33^,^34^ 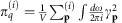

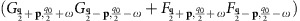
. 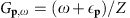 and *F*_**p**,*ω*_=Δ_**p**_/*Z* are the normal and anomalous Green's functions for the paired state, where 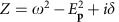 and *δ*→0^+^. 
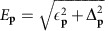
 is the quasiparticle energy and Δ_**p**_=*γ*_**p**_Δ is the *d*-wave gap function. It follows (Supplementary Note 1)


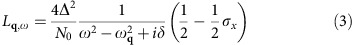


with the LM dispersion 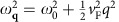. In this case, the LM is a coherent bosonic mode with an infinite lifetime. The LM is gapped, and its frequency at **q**=0 satisfies 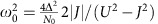. The ratio 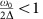 when 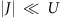.

### Phonon self-energy

**Figure 2:**
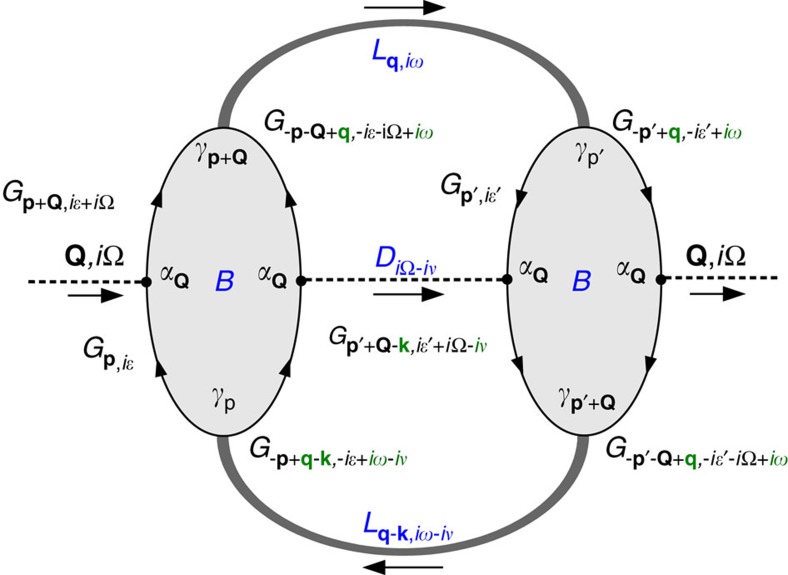
Feynman diagram of phonon self-energy. Dashed lines are phonon propagators (*D*) and thick grey lines are Leggett mode propagators (*L*). *B* is the effective interaction vertex between phonons and Leggett modes, which consists of a particle–particle electronic bubble (*G* is the electron Green's function). The incoming phonon is scattered forwardly to a nearby wavevector by the absorption and emission of the Leggett mode. Due to the separation of energy scales between the Leggett mode and the electron, all quantities in green are neglected.

The **k**-space separation of the two bands does not move the LM away from **q**=0, since this phase mode involves the transfer of zero momentum Cooper pairs between the sub-bands. Nonetheless it involves moving charges between the sub-bands. Absorption and emission of phonons with the appropriate wavevectors also causes a charge transfer, but now as single quasi-particles, between the two sub-bands. Therefore it is not unexpected that a coupling between these processes should exist. In particular, we find the coupling is largest in the temperature region *T*∼*T*_c_, where the LM drops to zero energy and becomes overdamped. To this end we consider the process outlined in Fig. 2, where an incoming phonon is scattered to a nearby phonon wavevector with emission and absorption of LM fluctuations. Such a process does not occur in standard superconductors but can exist here because of a soft overdamped LM for *T*_c_<*T*<*T*_o_. Below we summarize calculations of the phonon self-energy in two temperature regions.

**Figure 3:**
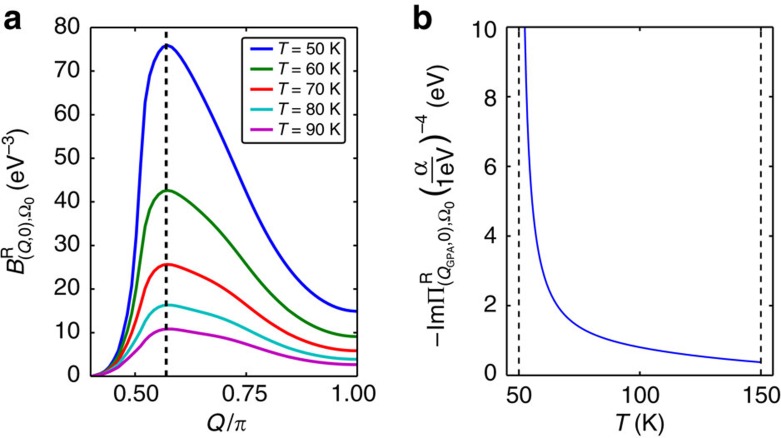
GPA in the fluctuation region. (**a**) Temperature and momentum dependence of the effective interaction vertex *B*, with the dashed line marking *Q*_GPA_. (**b**) The anomalous phonon damping. Parameters: Fermi velocity *v*_F_=500 meV, bare phonon frequency Ω_0_=10 meV, ratio between the Leggett mode frequency at **q**=0 and the superconducting gap 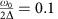, quasiparticle damping Γ^(e)^=0.5*T*+(0.3 meV^−1^)*T*^2^, and the long-range order temperature *T*_c_=50 K. We used an energy cutoff of ±100 meV around the Fermi surface, which does not affect the qualitative feature of the results.

First, we look at the phonon damping in the range of strong SC fluctuations, starting at the onset temperature *T*_o_ of the SC fluctuations down to the SC transition temperature *T*_c_. The expression for the phonon self-energy Π, corresponding to the Feynman diagram Fig. 2, follows


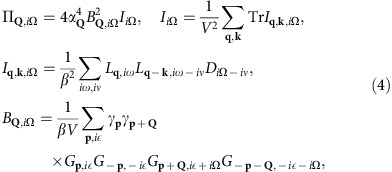


where *α*_**Q**_ is the electron-phonon coupling, *I*_**q**,**k**,*i*Ω_ is the frequency summation for the intermediate state (consisting of two LMs and one phonon), *B*_**Q**,*i*Ω_ is the effective interaction vertex between phonons and LMs, and 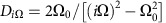 is the bare Green's function for phonons with an assumed flat dispersion Ω_**Q**_=Ω_0_. We have chosen the simplest form of the effective interaction, *B* and ignored damping due to quasiparticle excitations, keeping only 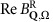, to concentrate on the phonon damping caused by the presence of a soft LM. The frequency summation in the expression for *B*^R^ is cast as an integral along the real axis (Supplementary Fig. 2), then the frequency and momentum integrals are carried out numerically (Supplementary Note 2). Note, Re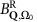 has a strong dependence on the phonon wavevector **Q** (see Fig. 3) and peaks at a wavevector joining the ends of the arcs, because the symmetry factors and the available phase space for the transition at this wavevector are both large. We checked that 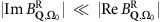 for the chosen parameters. Re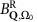 also shows a peak near **Q**=0 which will be discussed later.

The effective interaction vertex *B* involves an integral over the whole Brillouin zone and all frequencies, while the LM *L* is only well-defined for small momenta and frequencies. This leads to a separation of spatial and temporal scales and enables us to ignore all small wavevectors and frequencies (marked green in Fig. 2) in calculating *B*. Aided by the similarity with the Aslamazov-Larkin diagram^35^,^36^,^37^,^38^,^39^, we perform analytical calculation for 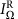. For 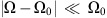, it follows (see Supplementary Note 2)


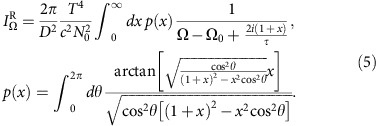


In this temperature region the on-shell values satisfy Re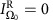 and Im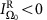. The temperature dependence of the imaginary part of the retarded phonon self-energy ImΠ^R^, that is, the phonon damping, is plotted in Fig. 3. The self-energy has a peak in momentum space at **Q**=(*Q*_GPA_, 0), near to the tip to tip wavevector between two sets of Fermi arcs. Because of the factor 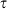 in equation (5), the temperature dependence shows anomalous behaviour at the long-range critical temperature *T*_c_, in agreement with the experiment^4^.

Below *T*_c_, there is a finite restoring force for inter-sub-band phase fluctuations and the LM develops a finite energy at **q**=0, which raises the energy of the intermediate state in Fig. 2. As a consequence the approximate resonant condition between the incoming phonon and the intermediate state with a scattered phonon and two LMs no longer holds, leading to a suppression of the phonon damping at low *T*. The GPA changes its form at *T*<*T*_c_ with strongly reduced damping. An anomaly in the phonon dispersion appears due to the virtual coupling to an excited intermediate state. Treating the low temperature behaviour at *T*=0, the factor from the intermediate state becomes


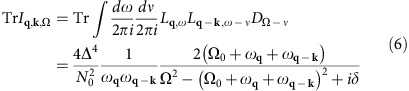


where 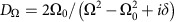. We conclude that in this region, the on-shell values satisfy 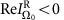 and 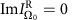, in agreement with the experiment for general *T*<*T*_c_ cases^4^.

At long wavelengths, **Q**∼0, the long-range nature of the Coulomb interaction suppresses the response in metals at low frequencies to any perturbation coupling to the total electronic density. The electron-phonon interaction introduced in equation (4) couples equally to both sub-bands and as a result the associated scattering processes are suppressed at **Q**∼0.

## Discussion

As we remarked earlier the transition into the pseudogap phase at the hole density *p*_c1_∼0.18 displays three strong anomalies simultaneously—an antinodal insulating energy gap leading to a breakup of the Fermi surface into four nodal pockets, a rapid expansion of the temperature range of superconducting fluctuations, and the appearance of a giant phonon anomaly in this temperature range. These phenomena are unique to the underdoped cuprates. Our aim here is to put forward a microscopic scenario, which explains the interrelation between these phenomena. The low energy Coulomb interaction must be strong to drive the partial truncation of the Fermi surface, which is a precursor to a full Mott gap at zero doping^22^,^23^. A weakness of our microscopic scenario is the need to assume a form for this effective Coulomb interaction. Our choice is guided by the evolution found by the functional renormalization group in the overdoped density region^26^,^27^. The persistence of *d*-wave symmetry even as the maximally gapped antinodal regions transform from a superconducting to an insulating gap, leads to the conditions for a low energy LM to emerge. As we discussed above, this assumption enables us to consistently explain all three anomalies. In particular we can explain the special temperature evolution of the GPA characterized by increasing damping as *T*→*T*_c_ from above, which abruptly changes to a GPA with vanishing damping but a dispersion anomaly at *T*<*T*_c_. Here we considered only zero magnetic field. The recent quantum oscillation experiments at high magnetic fields are consistently explained by a coherent orbit around all four arcs, which is intriguing^40^. It raises the question of the evolution of the LM with increasing magnetic field for future study. We note another interesting effect in a magnetic field is the enhanced Nernst effect in the expanded temperature range of superconducting fluctuations on passing into the pseudogap phase. This enhancement has been ascribed to strong phase fluctuations^41^ that agrees with our proposal.

## Additional information

**How to cite this article:** Liu, Y.-H. *et al.* Giant phonon anomaly associated with superconducting fluctuations in the pseudogap phase of cuprates. *Nat. Commun.* 7:10378 doi: 10.1038/ncomms10378 (2016).

## Supplementary Material

Supplementary Information Supplementary Figures 1-2, Supplementary Notes 1-2 and Supplementary References
