# Supplementary material for: Giant phonon anomaly associated with superconducting fluctuations in the pseudogap phase of cuprates
Source: Nat Commun. 2016 Jan 20;7:10378. doi: 10.1038/ncomms10378 (PMC4735821; doi:10.1038/ncomms10378)
Supplement: Supplementary Information — Supplementary Figures 1-2, Supplementary Notes 1-2 and Supplementary References [file ncomms10378-s1.pdf]

Supplementary Figures

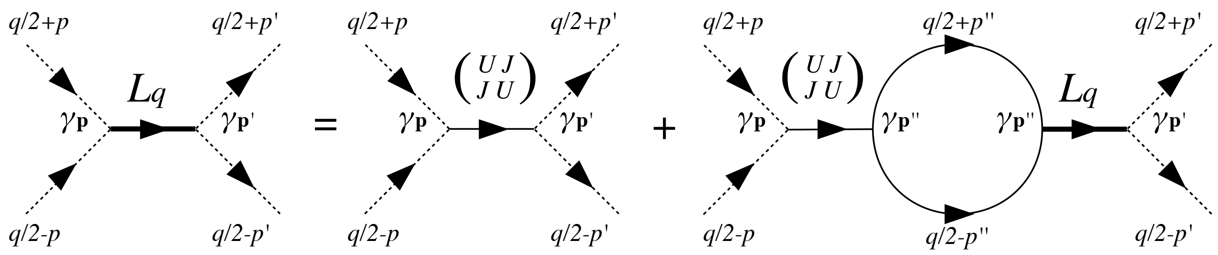

Supplementary Figure 1: **The Bethe-Salpeter equation for the Leggett mode propagator.** L_q is the Leggett mode propagator, $\gamma_{\mathbf{p}}$ is the d-wave symmetry factor, the matrix with elements U and V is the bare Cooper scattering in the subband space. p, p', p'' and q denote both momentum and frequency e.g. $q = (\mathbf{q}, iq_0)$.

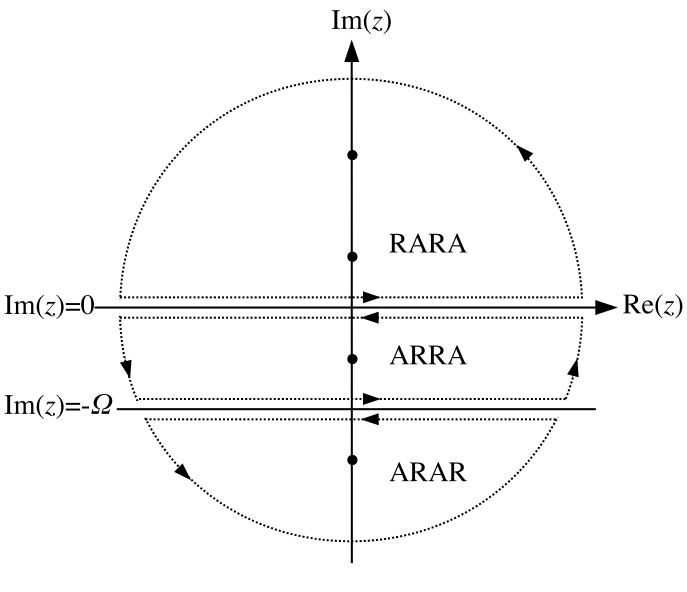

Supplementary Figure 2: **Contour integration in the complex z-plane.** Black dots are the fermionic frequencies to be summed over, bold black lines are the branch cuts of the integrand (where Ω is a bosonic frequency). R/A denotes retarded/advanced Green's functions in the integrand of Supplementary Equation (37).

Supplementary Note 1: Derivation of the Leggett mode

Fluctuation region

As mentioned in the main text, the propagator for superconducting fluctuations is derived by the following Bethe-Salpeter equation (see Supplementary Figure 1)

$$\begin{aligned} L_{pp'q} &= \gamma_{\mathbf{p}} L_q \gamma_{\mathbf{p}'} = \gamma_{\mathbf{p}} \begin{pmatrix} U & J \\ J & U \end{pmatrix} \gamma_{\mathbf{p}'} - \gamma_{\mathbf{p}} \begin{pmatrix} U & J \\ J & U \end{pmatrix} \begin{pmatrix} \pi_a(q) & 0 \\ 0 & \pi_b(q) \end{pmatrix} L_q \gamma_{\mathbf{p}'}, \\ \pi_i(q) &= \frac{1}{\beta V} \sum_{\mathbf{p}}^{(i)} \sum_{i\omega} \gamma_{\mathbf{p}}^2 G\left(\frac{\mathbf{q}}{2} + \mathbf{p}, iq_0 + i\omega\right) G\left(\frac{\mathbf{q}}{2} - \mathbf{p}, -i\omega\right), \end{aligned} \tag{1}$$

where $q = (\mathbf{q}, iq_0)$, and similarly for p and p'. Note that $U < 0$ and $J < 0$ are both attractive in our convention. The notation $\sum_{\mathbf{p}}^{(i)}$ means the summation over momentum space is restricted to the ith subband. Taking into account the strong damping of the quasiparticles in the underdoped regime, we assume a phenomenological form of the electron Green's function

$$\begin{aligned} G(\mathbf{p}, i\omega) &= \frac{1}{i\omega - \epsilon_{\mathbf{p}} + i\Gamma^{(\mathrm{e})}\mathrm{sgn}\omega}, \\ \Gamma^{(\mathrm{e})} &= aT + bT^2, \end{aligned} \tag{2}$$

where a quadratic temperature dependence of the quasiparticle damping rate is used according to previous transport studies [1]. To calculate π, we follow the general procedure of superconducting fluctuation theory [2, 3]. For any bosonic frequency $\omega_k > 0$, we have

$$\pi_i(\mathbf{q}, i\omega_k) \approx N_0 \sum_{n \geq 0} \left\langle \gamma_{\mathbf{p}}^2 \left[\frac{1}{n + \frac{1}{2} + \frac{\omega_k}{4\pi T} + \frac{\Gamma^{(\mathrm{e})}}{2\pi T}} - \frac{1}{(4\pi T)^2} \frac{(\mathbf{v}_{\mathrm{F}}(\mathbf{p}) \cdot \mathbf{q})^2}{\left(n + \frac{1}{2} + \frac{\omega_k}{4\pi T} + \frac{\Gamma^{(\mathrm{e})}}{2\pi T}\right)^3} \right] \right\rangle_i. \tag{3}$$

Here N_0 is the density of states per spin at the Fermi level in one subband, $\mathbf{v}_{\mathrm{F}}(\mathbf{p})$ is the Fermi velocity at momentum $\mathbf{p}$. We use $\langle \ldots \rangle_i$ to denote the average over the Fermi surface of subband i. A normalized d-wave symmetry factor $\gamma_{\mathbf{p}} = \sqrt{2} \cos 2\theta_{\mathbf{p}}$ is chosen, where $\theta_{\mathbf{p}}$ is the angle measured from the center of the arc and $\langle \gamma_{\mathbf{p}}^2 \rangle_i = 1$. Introducing a large bosonic frequency $\omega_{\mathrm{c}} = 2n_{\max}\pi T$ to cut off the summation of the first term (which is logarithmic divergent), it follows

$$\begin{aligned} \sum_{n \geq 0}^{n_{\max}} \frac{1}{n + \frac{1}{2} + \frac{\omega_k}{4\pi T} + \frac{\Gamma^{(\mathrm{e})}}{2\pi T}} &= \psi\left(\frac{1}{2} + \frac{\omega_k}{4\pi T} + \frac{\Gamma^{(\mathrm{e})}}{2\pi T} + \frac{\omega_{\mathrm{c}}}{2\pi T}\right) - \psi\left(\frac{1}{2} + \frac{\omega_k}{4\pi T} + \frac{\Gamma^{(\mathrm{e})}}{2\pi T}\right), \\ \sum_{n \geq 0} \frac{1}{\left(n + \frac{1}{2} + \frac{\omega_k}{4\pi T} + \frac{\Gamma^{(\mathrm{e})}}{2\pi T}\right)^3} &= -\frac{1}{2}\psi''\left(\frac{1}{2} + \frac{\omega_k}{4\pi T} + \frac{\Gamma^{(\mathrm{e})}}{2\pi T}\right), \end{aligned} \tag{4}$$

where the special function $\psi(x) = d[\log \Gamma(x)]/dx$ is the digamma function. The analytic continuation to real frequency is taken by $\omega_k \to -i\omega$, which leads to

$$\begin{aligned} \frac{1}{N_0} \pi_i^{\mathrm{R}}(\mathbf{q}, \omega) &= \psi\left(\frac{1}{2} - \frac{i\omega}{4\pi T} + \frac{a + bT}{2\pi} + \frac{\omega_{\mathrm{c}}}{2\pi T}\right) - \psi\left(\frac{1}{2} - \frac{i\omega}{4\pi T} + \frac{a + bT}{2\pi}\right) \\ &+ \frac{1}{2(4\pi T)^2} \psi''\left(\frac{1}{2} - \frac{i\omega}{4\pi T} + \frac{a + bT}{2\pi}\right) \left\langle \gamma_{\mathbf{p}}^2 (\mathbf{v}_{\mathrm{F}}(\mathbf{p}) \cdot \mathbf{q})^2 \right\rangle_i. \end{aligned} \tag{5}$$

The superconducting ordering temperature T_{c}, at which the fluctuation propagator $L^{\mathrm{R}}(\mathbf{q} = 0, \omega = 0)$ diverges, is given by

$$T_{\mathrm{c}} = \frac{\omega_{\mathrm{c}}}{2\pi} \exp\left[-\psi\left(\frac{1}{2} + \frac{a + bT_{\mathrm{c}}}{2\pi}\right)\right] \exp\left(-\frac{1}{N_0 |U + J|}\right). \tag{6}$$

Note that finite damping $a, b > 0$ suppresses T_{c}. When $a = b = 0$, the above equation reduces to the conventional BCS equation $T_{\mathrm{c}} = 1.13\omega_{\mathrm{c}} \exp\left[-1/\left(N_0 \left|V_{\mathrm{max}}\right|\right)\right]$, where V_{max} is the most attractive interaction, in our two subband case it is the eigenvalue $U + J$ of the bare interaction vertex. In terms of T_{c}, we could rewrite the bubble π as

$$\begin{aligned} \frac{1}{N_0}\pi_i^{\mathrm{R}}(\mathbf{q},\omega) &\approx -\frac{1}{N_0(U+J)} - \epsilon + \frac{1}{4\pi T}\psi'\left(\frac{1}{2} + \frac{a+bT_{\mathrm{c}}}{2\pi}\right)\left[i\omega - 2bT\left(T - T_{\mathrm{c}}\right)\right] \\ &+ \frac{1}{2(4\pi T)^2}\psi''\left(\frac{1}{2} + \frac{a+bT}{2\pi}\right)\left\langle \gamma_{\mathbf{p}}^2 \left(\mathbf{v}_{\mathrm{F}}(\mathbf{p})\cdot\mathbf{q}\right)^2\right\rangle_i, \end{aligned} \tag{7}$$

where $\epsilon = \log\left(T/T_{\mathrm{c}}\right)$ is the reduced temperature.

Solving the matrix Bethe-Salpeter equation for L^{R} (Supplementary Equation (1)), one gets

$$L^{\mathrm{R}}(\mathbf{q},\omega) = \frac{\begin{pmatrix} U + \left(U^2 - J^2\right)\pi_b^{\mathrm{R}}(\mathbf{q},\omega) & J \\ J & U + \left(U^2 - J^2\right)\pi_a^{\mathrm{R}}(\mathbf{q},\omega) \end{pmatrix}}{\left(U^2 - J^2\right)\pi_a^{\mathrm{R}}(\mathbf{q},\omega)\pi_b^{\mathrm{R}}(\mathbf{q},\omega) + U\left[\pi_a^{\mathrm{R}}(\mathbf{q},\omega) + \pi_b^{\mathrm{R}}(\mathbf{q},\omega)\right] + 1}. \tag{8}$$

If we make a further isotropic simplification by averaging over the direction of $\mathbf{q}$ such that $\pi_a^{\mathrm{R}} = \pi_b^{\mathrm{R}} = \pi^{\mathrm{R}}$, then

$$L^{\mathrm{R}} = \frac{\begin{pmatrix} U + \left(U^2 - J^2\right)\pi^{\mathrm{R}} & J \\ J & U + \left(U^2 - J^2\right)\pi^{\mathrm{R}} \end{pmatrix}}{\left[(U+J)\pi^{\mathrm{R}} + 1\right]\left[(U-J)\pi^{\mathrm{R}} + 1\right]} = \left[\frac{P_+}{\pi^{\mathrm{R}} + \frac{1}{U+J}} + \frac{P_-}{\pi^{\mathrm{R}} + \frac{1}{U-J}}\right], \tag{9}$$

where the two projectors in the nominators are

$$P_\pm = \left(\frac{1}{2} \pm \frac{1}{2}\sigma_x\right). \tag{10}$$

They satisfy $P_\pm^2 = P_\pm$ and $P_+P_- = 0$. Physically, P_+ projects into the subspace of in-phase fluctuations, and P_- into that of out-of-phase fluctuations. The position of the pole in P_+ is located at $\mathbf{q} = \omega = 0$ when $T = T_{\mathrm{c}}$. Thus it is the fluctuating version of the Anderson-mode pole (with in-phase nature and gapless nature). In the presence of a local superconducting order parameter, the in-phase phase fluctuation at long wavelengths is associated with the total charge density modulation, which is prohibited by the long-range Coulomb interaction. As a result we are mainly interested in the out-of-phase fluctuations i.e. the fluctuating version of the Leggett mode with nominator P_-

$$L^{\mathrm{R}}(\mathbf{q},\omega) = \frac{T}{cN_0}\frac{P_-}{i\omega - \tau^{-1} - Dq^2}, \tag{11}$$

where the constant $c = \frac{1}{4\pi}\psi'\left[\frac{1}{2} + \frac{1}{2\pi}\left(a + bT_{\mathrm{c}}\right)\right]$. The above propagator has the form of an overdamped bosonic mode. The total relaxation time τ and diffusion constant D are defined by the following equations

$$\begin{aligned} \tau^{-1} &= \frac{T}{c}\log\frac{T}{T_{\mathrm{c}}} + 2bT\left(T - T_{\mathrm{c}}\right) + \frac{T}{cN_0}\frac{2|J|}{U^2 - J^2}, \\ Dq^2 &= -\frac{1}{8\pi T}\frac{\psi''\left[\frac{1}{2} + \frac{1}{2\pi}(a + bT)\right]}{\psi'\left[\frac{1}{2} + \frac{1}{2\pi}\left(a + bT_{\mathrm{c}}\right)\right]}\left\langle \gamma_{\mathbf{p}}^2\left(\mathbf{v}_{\mathrm{F}}(\mathbf{p})\cdot\mathbf{q}\right)^2\right\rangle \\ &= -\frac{v_{\mathrm{F}}^2}{16\pi T}\frac{\psi''\left[\frac{1}{2} + \frac{1}{2\pi}(a + bT)\right]}{\psi'\left[\frac{1}{2} + \frac{1}{2\pi}\left(a + bT_{\mathrm{c}}\right)\right]}q^2, \end{aligned} \tag{12}$$

where in the expression for D, the symbol $\langle \ldots \rangle$ means averaging over both the directions of $\mathbf{p}$ and $\mathbf{q}$, and the result is independent on the subband index i. From now on we denote the total damping of Leggett mode to be

$$\Gamma_{\mathbf{q}}^{(\mathrm{LM})} = \tau^{-1} + Dq^2, \tag{13}$$

which leads to the simple expression used in the main text

$$L^{\mathrm{R}}(\mathbf{q},\omega) = \frac{T}{cN_0}\frac{1}{i\omega - \Gamma_{\mathbf{q}}^{(\mathrm{LM})}}\left(\frac{1}{2} - \frac{1}{2}\sigma_x\right). \tag{14}$$

Ordered region $T=0$

In this case, the structure of the Bethe-Salpeter equation is unchanged, but the electronic bubble receives another contribution from the anomalous Green's function due to the superconducting order [4–6]. Also the Green's functions change form in the superconducting phase, $G_p = (ip_0 + \epsilon_{\mathbf{p}}) / \left[(ip_0)^2 - E_{\mathbf{p}}^2\right]$ and $F_p = \Delta_{\mathbf{p}} / \left[(ip_0)^2 - E_{\mathbf{p}}^2\right]$, with $E_{\mathbf{p}} = \sqrt{\epsilon_{\mathbf{p}}^2 + \Delta_{\mathbf{p}}^2}$ the Bogoliubov quasiparticle energy. We generalize the derivation by Leggett for s-wave superconductors [4] to the d-wave case. In the following equations, the $\mathbf{q}$-dependent quantities are $\epsilon_{\mathbf{p}\pm\frac{\mathbf{q}}{2}}$, $E_{\mathbf{p}\pm\frac{\mathbf{q}}{2}}$, and $\Delta_{\mathbf{p}\pm\frac{\mathbf{q}}{2}}$. Because $|\mathbf{q}| \ll 2\pi$ is small and $|\partial_{\mathbf{p}}\epsilon_{\mathbf{p}}| \gg |\partial_{\mathbf{p}}\Delta_{\mathbf{p}}|$, we set $\Delta_{\mathbf{p}\pm\frac{\mathbf{q}}{2}} \approx \Delta_{\mathbf{p}} = \gamma_{\mathbf{p}}\Delta$. Then it follows

$$\begin{aligned}\pi_i(q) &= \frac{1}{\beta V}\sum_{\mathbf{p}}^{(i)}\sum_{ip_0}\gamma_{\mathbf{p}}^2\left[G\left(\frac{\mathbf{q}}{2}+\mathbf{p}, iq_0+ip_0\right)G\left(\frac{\mathbf{q}}{2}-\mathbf{p}, -ip_0\right)+F\left(\frac{\mathbf{q}}{2}+\mathbf{p}, iq_0+ip_0\right)F\left(\frac{\mathbf{q}}{2}-\mathbf{p}, -ip_0\right)\right]\\ &\approx N_0\int d\epsilon_{\mathbf{p}}\left\langle\gamma_{\mathbf{p}}^2\frac{\tanh\frac{E_{\mathbf{p}}}{2T}}{2E_{\mathbf{p}}}\right\rangle_i - N_0\left\langle\frac{(iq_0)^2-(\mathbf{v}_{\mathrm{F}}(\mathbf{p})\cdot\mathbf{q})^2}{2\Delta^2}\frac{\Delta_{\mathbf{p}}^2}{2E_{\mathbf{p}}}\frac{\partial}{\partial E_{\mathbf{p}}}\left(\int d\epsilon_{\mathbf{p}}\frac{\tanh\frac{E_{\mathbf{p}}}{2T}}{2E_{\mathbf{p}}}\right)\right\rangle_i.\end{aligned} \tag{15}$$

Here we have taken the leading order term of q and used the identity

$$\int d\epsilon_{\mathbf{p}}\sum_{ip_0}\frac{\frac{1}{2}iq_0+ip_0+\epsilon_{\mathbf{p}}}{\left[(iq_0+ip_0)^2-E_{\mathbf{p}+\frac{\mathbf{q}}{2}}^2\right]\left[(ip_0)^2-E_{\mathbf{p}-\frac{\mathbf{q}}{2}}^2\right]} = 0, \tag{16}$$

because the integrand is odd under $\epsilon_{\mathbf{p}} \to -\epsilon_{\mathbf{p}}$ and $ip_0 \to -ip_0 - iq_0$. In zero temperature, the first term in the last line of Supplementary Equation (15) becomes

$$N_0\int d\epsilon_{\mathbf{p}}\left\langle\frac{\gamma_{\mathbf{p}}^2}{2E_{\mathbf{p}}}\right\rangle_i = -\frac{1}{U+J}, \tag{17}$$

which follows from the gap equation. The energy integral in the second term is

$$\frac{\Delta_{\mathbf{p}}^2}{2E_{\mathbf{p}}}\frac{\partial}{\partial E_{\mathbf{p}}}\int d\epsilon_{\mathbf{p}}\frac{1}{2E_{\mathbf{p}}} = -\frac{1}{4}\int d\epsilon_{\mathbf{p}}\frac{\Delta_{\mathbf{p}}^2}{E_{\mathbf{p}}^3} = -\frac{1}{4}\int d\epsilon_{\mathbf{p}}\frac{\partial}{\partial\epsilon_{\mathbf{p}}}\left(\frac{\epsilon_{\mathbf{p}}}{E_{\mathbf{p}}}\right) = -\frac{1}{2}. \tag{18}$$

After a Wick rotation $iq_0 \to \omega$ and averaging the direction of $\mathbf{q}$, the zero temperature bubble reads

$$\pi(\mathbf{q},\omega) = -\frac{1}{U+J} + \frac{N_0}{4\Delta^2}\left(\omega^2 - \frac{1}{2}v_{\mathrm{F}}^2q^2\right). \tag{19}$$

Then

$$L(\mathbf{q},\omega) = \frac{1}{\pi(\mathbf{q},\omega)+\frac{1}{U-J}}\left(\frac{1}{2}-\frac{1}{2}\sigma_x\right) = \frac{4\Delta^2}{N_0}\frac{1}{\omega^2-\omega_{\mathbf{q}}^2+i\delta}\left(\frac{1}{2}-\frac{1}{2}\sigma_x\right), \tag{20}$$

where the dispersion of the Leggett mode is

$$\begin{aligned}\omega_{\mathbf{q}}^2 &= \omega_0^2 + \frac{1}{2}v_{\mathrm{F}}^2q^2,\\ \omega_0^2 &= \frac{4\Delta^2}{N_0}\frac{2|J|}{U^2-J^2}.\end{aligned} \tag{21}$$

Supplementary Note 2: Calculation of the phonon self energy

Summation of subband indices

In the main text, the full expression for the phonon self energy is written as

$$\begin{aligned}\Pi^{\mathrm{R}}(\mathbf{Q},\Omega) &= 4\left[\alpha(\mathbf{Q})\right]^4\left[B^{\mathrm{R}}(\mathbf{Q},\Omega)\right]^2 I^{\mathrm{R}}(\Omega),\\ I^{\mathrm{R}}(\Omega) &= \frac{1}{V^2}\sum_{\mathbf{q},\mathbf{k}}\mathrm{Tr}I^{\mathrm{R}}(\mathbf{q},\mathbf{k},\Omega).\end{aligned} \tag{22}$$

The meaning of the trace (Tr) in Supplementary Equation (22) is explained as follows. We are interested in the case of $|\mathbf{Q}|$ being large enough to lead to inter-subband transitions. In this case the incoming and outgoing Cooper pairs in the diagram for B are from two subbands, thus B is considered to be off-diagonal in the subband space. The fluctuation propagators, on the other hand, contain intra-subband and inter-subband components. As a result, all possibilities of the arrangements of subband indices sum up to a trace over the product of the two fluctuation propagators

$$\mathrm{Tr}\left[\begin{pmatrix}0 & B\\ B & 0\end{pmatrix}\begin{pmatrix}L^{(1)}_{\mathrm{intra}} & L^{(1)}_{\mathrm{inter}}\\ L^{(1)}_{\mathrm{inter}} & L^{(1)}_{\mathrm{intra}}\end{pmatrix}\begin{pmatrix}0 & B\\ B & 0\end{pmatrix}\begin{pmatrix}L^{(2)}_{\mathrm{intra}} & L^{(2)}_{\mathrm{inter}}\\ L^{(2)}_{\mathrm{inter}} & L^{(2)}_{\mathrm{intra}}\end{pmatrix}\right] = B^2\mathrm{Tr}\left[\begin{pmatrix}L^{(1)}_{\mathrm{intra}} & L^{(1)}_{\mathrm{inter}}\\ L^{(1)}_{\mathrm{inter}} & L^{(1)}_{\mathrm{intra}}\end{pmatrix}\begin{pmatrix}L^{(2)}_{\mathrm{intra}} & L^{(2)}_{\mathrm{inter}}\\ L^{(2)}_{\mathrm{inter}} & L^{(2)}_{\mathrm{intra}}\end{pmatrix}\right]. \tag{23}$$

Intermediate state in fluctuation region

We use the spectral decomposition for the fluctuation propagator L and the bare phonon Green's function D as follows

$$\begin{aligned}L(\mathbf{q},i\omega) &= \int\frac{dx}{2\pi}\frac{-2\mathrm{Im}L^{\mathrm{R}}(\mathbf{q},x)}{i\omega-x},\\ D(i\omega) &= \int\frac{dx}{2\pi}\frac{-2\mathrm{Im}D^{\mathrm{R}}(x)}{i\omega-x},\end{aligned} \tag{24}$$

where the phonon is assumed to be dispersionless for simplicity, which is justified because we are only interested in large phonon momentum. The frequency summation of the intermediate state becomes

$$\begin{aligned}I(\mathbf{q},\mathbf{k},i\Omega) &= \frac{1}{\beta^2}\sum_{i\omega,i\nu}L(\mathbf{q},i\omega)L(\mathbf{q}-\mathbf{k},i\omega-i\nu)D(i\Omega-i\nu)\\ &= -\frac{1}{\pi^3}\int dxdydz\,\mathrm{Im}L^{\mathrm{R}}(\mathbf{q},x)\,\mathrm{Im}L^{\mathrm{R}}(\mathbf{q}-\mathbf{k},y)\,\mathrm{Im}D^{\mathrm{R}}(z)\\ &\quad\times\frac{1}{\beta^2}\sum_{i\omega,i\nu}\frac{1}{i\omega-x}\frac{1}{i\omega-i\nu-y}\frac{1}{i\Omega-i\nu-z},\end{aligned} \tag{25}$$

where the last line could be carried out explicitly as follows

$$\frac{1}{\beta^2}\sum_{i\omega,i\nu}\frac{1}{i\omega-x}\frac{1}{i\omega-i\nu-y}\frac{1}{i\Omega-i\nu-z} = \frac{1}{4}\frac{\left(-\coth\frac{x}{2T}+\coth\frac{y}{2T}\right)\left(\coth\frac{x-y}{2T}+\coth\frac{z}{2T}\right)}{i\Omega-x+y-z}. \tag{26}$$

We then perform the analytic continuation of the external frequency $i\Omega\to\Omega+i\delta$, and take the imaginary part of the whole expression. Now the integral contains two delta-functions

$$\begin{aligned}\mathrm{Im}\frac{1}{\Omega-x+y-z+i\delta} &= -\pi\delta(\Omega-x+y-z),\\ \mathrm{Im}D^{\mathrm{R}}(z) &= -\pi\delta(z-\Omega_0)+\pi\delta(z+\Omega_0),\end{aligned} \tag{27}$$

where Ω_0 is the phonon frequency. $\mathrm{Im}I^{\mathrm{R}}(\mathbf{q},\mathbf{k},\Omega)$ could now be written as a one-dimensional integral

$$\begin{aligned}
\mathrm{Im} I^{\mathrm{R}}(\mathbf{q},\mathbf{k},\Omega) &= -\frac{1}{\pi}\int dx\, \mathrm{Im} L^{\mathrm{R}}(\mathbf{q},x)\, \mathrm{Im} L^{\mathrm{R}}(\mathbf{q}-\mathbf{k},x-\Omega+\Omega_0) \\
&\times \frac{1}{4}\left(-\coth\frac{x}{2T}+\coth\frac{x-\Omega+\Omega_0}{2T}\right)\left(\coth\frac{\Omega-\Omega_0}{2T}+\coth\frac{\Omega_0}{2T}\right) \\
&- \{\Omega_0 \to -\Omega_0\}. \qquad (28)
\end{aligned}$$

We are interested in the region where $|\Omega-\Omega_0| \ll T$, thus we denote $\delta\Omega = \Omega-\Omega_0$. The frequencies for the two Leggett modes in the integrand are x and $x-\delta\Omega$. Because the Leggett mode has spectral weight only in a small frequency range near $x=0$, we ignore the $\{\Omega_0 \to -\Omega_0\}$ term in the last line. When $\Omega \to \Omega_0$, $\left|\coth\frac{\Omega-\Omega_0}{2T}\right| \gg \left|\coth\frac{\Omega_0}{2T}\right|$, so we also neglect the $\coth\frac{\Omega_0}{2T}$ term. After these simplifications, we arrive at

$$\mathrm{Im} I^{\mathrm{R}}(\mathbf{q},\mathbf{k},\delta\Omega) = \frac{1}{4\pi}\coth\frac{\delta\Omega}{2T}\int dx\, \mathrm{Im} L^{\mathrm{R}}(\mathbf{q},x)\, \mathrm{Im} L^{\mathrm{R}}(\mathbf{q}-\mathbf{k},x-\delta\Omega)\left(\coth\frac{x}{2T}-\coth\frac{x-\delta\Omega}{2T}\right). \qquad (29)$$

The above result is to be summed over $\mathbf{q}$ and $\mathbf{k}$, which allows us to make shifts, so that

$$\begin{aligned}
&\mathrm{Tr}\,\mathrm{Im} I^{\mathrm{R}}(\mathbf{q},\mathbf{k},\delta\Omega) \\
\to\ & \frac{1}{4\pi}\coth\frac{\delta\Omega}{2T}\int dx\, \mathrm{Tr}\left[\mathrm{Im} L^{\mathrm{R}}\left(\mathbf{q}+\frac{\mathbf{k}}{2},x+\frac{\delta\Omega}{2}\right)\mathrm{Im} L^{\mathrm{R}}\left(\mathbf{q}-\frac{\mathbf{k}}{2},x-\frac{\delta\Omega}{2}\right)\right]\left(\coth\frac{x+\frac{\delta\Omega}{2}}{2T}-\coth\frac{x-\frac{\delta\Omega}{2}}{2T}\right) \\
\approx\ & -T^2\left(\frac{T}{cN_0}\right)^2\left(\frac{1}{\Gamma^{(\mathrm{LM})}_{\mathbf{q}+\frac{\mathbf{k}}{2}}}+\frac{1}{\Gamma^{(\mathrm{LM})}_{\mathbf{q}-\frac{\mathbf{k}}{2}}}\right)\frac{1}{\delta\Omega^2+\left(\Gamma^{(\mathrm{LM})}_{\mathbf{q}+\frac{\mathbf{k}}{2}}+\Gamma^{(\mathrm{LM})}_{\mathbf{q}-\frac{\mathbf{k}}{2}}\right)^2} \\
=\ & -\frac{2T^4}{c^2N_0^2}\frac{\tau^{-1}+D\left(q^2+k^2/4\right)}{\left\{\left[\tau^{-1}+D\left(q^2+k^2/4\right)\right]^2-D^2q^2k^2\cos^2\theta\right\}\left\{\delta\Omega^2+4\left[\tau^{-1}+D\left(q^2+k^2/4\right)\right]^2\right\}}. \qquad (30)
\end{aligned}$$

In the derivation we have used $\coth x \approx 1/x$ when $x<1$. Here θ is the angle between $\mathbf{q}$ and $\mathbf{k}$. The momentum integral then follows

$$\begin{aligned}
&\frac{1}{V^2}\sum_{\mathbf{q},\mathbf{k}}\frac{\tau^{-1}+D\left(q^2+k^2/4\right)}{\left\{\left[\tau^{-1}+D\left(q^2+k^2/4\right)\right]^2-D^2q^2k^2\cos^2\theta\right\}\left\{\delta\Omega^2+4\left[\tau^{-1}+D\left(q^2+k^2/4\right)\right]^2\right\}} \\
=\ & 2\pi\int_0^\infty dx\int_0^\infty dy\int_0^{2\pi} d\theta\frac{\tau^{-1}+D(x+y)}{\left\{\left[\tau^{-1}+D(x+y)\right]^2-4D^2xy\cos^2\theta\right\}\left\{\delta\Omega^2+4\left[\tau^{-1}+D(x+y)\right]^2\right\}} \\
=\ & 2\pi\int_0^\infty dx\int_0^{2\pi} d\theta\int_{-x}^{+x} dy\frac{\tau^{-1}+\sqrt{2}Dx}{\left\{\left(\tau^{-1}+\sqrt{2}Dx\right)^2-2D^2\left(x^2-y^2\right)\cos^2\theta\right\}\left\{\delta\Omega^2+4\left(\tau^{-1}+\sqrt{2}Dx\right)^2\right\}} \\
=\ & \frac{\pi\tau}{D^2}\int_0^\infty dx\int_0^{2\pi} d\theta\frac{\arctan\left[\sqrt{\frac{\cos^2\theta}{(1+x)^2-x^2\cos^2\theta}}x\right]}{\sqrt{\cos^2\theta\left[(1+x)^2-x^2\cos^2\theta\right]}}\frac{2(1+x)}{\tau^2\delta\Omega^2+4(1+x)^2}. \qquad (31)
\end{aligned}$$

Defining a nonnegative weight function

$$p(x)=\int_0^{2\pi} d\theta\frac{\arctan\left[\sqrt{\frac{\cos^2\theta}{(1+x)^2-x^2\cos^2\theta}}x\right]}{\sqrt{\cos^2\theta\left[(1+x)^2-x^2\cos^2\theta\right]}}, \qquad (32)$$

we then have

$$\mathrm{Im} I^{\mathrm{R}}(\delta\Omega) = \frac{2\pi}{D^2}\frac{T^4}{c^2N_0^2}\int_0^\infty dx\, p(x)\,\mathrm{Im}\frac{1}{\delta\Omega+\frac{2i(1+x)}{\tau}}. \qquad (33)$$

Our result is valid for $|\delta\Omega| \ll T$. If we extrapolate the above result to the whole range of $\delta\Omega$, then the Kramers-Kronig relations lead to

$$I^{\mathrm{R}}(\delta\Omega) = \frac{2\pi}{D^2}\frac{T^4}{c^2 N_0^2}\int_0^\infty dx\, p(x)\,\frac{1}{\delta\Omega + \frac{2i(1+x)}{\tau}}. \tag{34}$$

Intermediate state in ordered region $T = 0$

At $T = 0$, the Anderson mode is pushed towards the plasma frequency and is decoupled from the low-energy physics. The only remaining low-lying collective mode is then the Leggett mode. We rewrite the zero-temperature form of the Leggett mode and the phonon Green's function as follows

$$\begin{aligned} L(\mathbf{q},\omega) &= \frac{4\Delta^2}{N_0}\frac{1}{2\omega_{\mathbf{q}}}\left(\frac{1}{\omega-\omega_{\mathbf{q}}+i\delta} - \frac{1}{\omega+\omega_{\mathbf{q}}-i\delta}\right)\left(\frac{1}{2}-\frac{1}{2}\sigma_x\right), \\ D(\Omega) &= \frac{1}{\Omega-\Omega_0+i\delta} - \frac{1}{\Omega+\Omega_0-i\delta}. \end{aligned} \tag{35}$$

We then carry out the frequency integral of the intermediate state by the method of residues

$$\begin{aligned} &\mathrm{Tr} I(\mathbf{q},\mathbf{k},\Omega) \\ =\ &\mathrm{Tr}\int \frac{d\omega}{2\pi i}\frac{d\nu}{2\pi i} L(\mathbf{q},\omega) L(\mathbf{q}-\mathbf{k},\omega-\nu) D(\Omega-\nu) \\ =\ &\frac{4\Delta^4}{N_0^2}\frac{1}{\omega_{\mathbf{q}}\omega_{\mathbf{q}-\mathbf{k}}}\int \frac{d\omega}{2\pi i}\frac{d\nu}{2\pi i}\left(\frac{1}{\omega-\omega_{\mathbf{q}}+i\delta} - \frac{1}{\omega+\omega_{\mathbf{q}}-i\delta}\right) \\ &\times\left(\frac{1}{\omega-\nu-\omega_{\mathbf{q}-\mathbf{k}}+i\delta} - \frac{1}{\omega-\nu+\omega_{\mathbf{q}-\mathbf{k}}-i\delta}\right)\left(\frac{1}{\Omega-\nu-\Omega_0+i\delta} - \frac{1}{\Omega-\nu+\Omega_0-i\delta}\right) \\ =\ &\frac{4\Delta^4}{N_0^2}\frac{1}{\omega_{\mathbf{q}}\omega_{\mathbf{q}-\mathbf{k}}}\left[\frac{1}{\Omega-(\Omega_0+\omega_{\mathbf{q}}+\omega_{\mathbf{q}-\mathbf{k}})+i\delta} - \frac{1}{\Omega+(\Omega_0+\omega_{\mathbf{q}}+\omega_{\mathbf{q}-\mathbf{k}})-i\delta}\right]. \end{aligned} \tag{36}$$

Numerical calculation for B in the fluctuation region

With the damping effect, the electron Green's function $G(\mathbf{k}, i\omega \to z)$ has a branch-cut on the real-axis $\mathrm{Im}z = 0$. In this case, we do the frequency summation by expanding the contour integral of z around the imaginary axis to infinity, but avoiding the branch-cuts of the integrand as follows (Supplementary Figure 2)

$$\begin{aligned} &\frac{1}{\beta}\sum_{i\omega} G(\mathbf{k},i\omega) G(-\mathbf{k},-i\omega) G(\mathbf{k}+\mathbf{Q},i\omega+i\Omega) G(-\mathbf{k}-\mathbf{Q},-i\omega-i\Omega) \\ =\ &\frac{1}{4\pi i}\oint dz \tanh\frac{z}{2T} G(\mathbf{k},z) G(\mathbf{k},-z) G(\mathbf{k}+\mathbf{Q},z+i\Omega) G(\mathbf{k}+\mathbf{Q},-z-i\Omega) \\ =\ &\frac{1}{4\pi i}\int dx \tanh\frac{x}{2T} G^{\mathrm{R}}(\mathbf{k},x) G^{\mathrm{A}}(\mathbf{k},-x) G^{\mathrm{R}}(\mathbf{k}+\mathbf{Q},x+i\Omega) G^{\mathrm{A}}(\mathbf{k}+\mathbf{Q},-x-i\Omega) \\ -\ &\frac{1}{4\pi i}\int dx \tanh\frac{x}{2T} G^{\mathrm{A}}(\mathbf{k},x) G^{\mathrm{R}}(\mathbf{k},-x) G^{\mathrm{R}}(\mathbf{k}+\mathbf{Q},x+i\Omega) G^{\mathrm{A}}(\mathbf{k}+\mathbf{Q},-x-i\Omega) \\ +\ &\frac{1}{4\pi i}\int dx \tanh\frac{x}{2T} G^{\mathrm{A}}(\mathbf{k},x-i\Omega) G^{\mathrm{R}}(\mathbf{k},-x+i\Omega) G^{\mathrm{R}}(\mathbf{k}+\mathbf{Q},x) G^{\mathrm{A}}(\mathbf{k}+\mathbf{Q},-x) \\ -\ &\frac{1}{4\pi i}\int dx \tanh\frac{x}{2T} G^{\mathrm{A}}(\mathbf{k},x-i\Omega) G^{\mathrm{R}}(\mathbf{k},-x+i\Omega) G^{\mathrm{A}}(\mathbf{k}+\mathbf{Q},x) G^{\mathrm{R}}(\mathbf{k}+\mathbf{Q},-x). \end{aligned} \tag{37}$$

In the above equations, z is a complex number and x is a real number. The analytic continuation of the phonon frequency $i\Omega \to \Omega + i\delta$ could then be performed to lead to the following form

$$
\begin{aligned}
& B^R(\mathbf{Q},\Omega) \\
= & \frac{1}{4\pi i}\int\frac{d\mathbf{k}}{(2\pi)^2}\gamma_{\mathbf{k}}\gamma_{\mathbf{k}+\mathbf{Q}}\int dx\,\tanh\frac{x}{2T}\,G^{\mathrm{R}}(\mathbf{k},x)\,G^{\mathrm{A}}(\mathbf{k},-x)\,G^{\mathrm{R}}(\mathbf{k}+\mathbf{Q},x+\Omega)\,G^{\mathrm{A}}(\mathbf{k}+\mathbf{Q},-x-\Omega) \\
- & \frac{1}{4\pi i}\int\frac{d\mathbf{k}}{(2\pi)^2}\gamma_{\mathbf{k}}\gamma_{\mathbf{k}+\mathbf{Q}}\int dx\,\tanh\frac{x}{2T}\,G^{\mathrm{A}}(\mathbf{k},x)\,G^{\mathrm{R}}(\mathbf{k},-x)\,G^{\mathrm{R}}(\mathbf{k}+\mathbf{Q},x+\Omega)\,G^{\mathrm{A}}(\mathbf{k}+\mathbf{Q},-x-\Omega) \\
+ & \frac{1}{4\pi i}\int\frac{d\mathbf{k}}{(2\pi)^2}\gamma_{\mathbf{k}}\gamma_{\mathbf{k}+\mathbf{Q}}\int dx\,\tanh\frac{x+\Omega}{2T}\,G^{\mathrm{A}}(\mathbf{k},x)\,G^{\mathrm{R}}(\mathbf{k},-x)\,G^{\mathrm{R}}(\mathbf{k}+\mathbf{Q},x+\Omega)\,G^{\mathrm{A}}(\mathbf{k}+\mathbf{Q},-x-\Omega) \\
- & \frac{1}{4\pi i}\int\frac{d\mathbf{k}}{(2\pi)^2}\gamma_{\mathbf{k}}\gamma_{\mathbf{k}+\mathbf{Q}}\int dx\,\tanh\frac{x+\Omega}{2T}\,G^{\mathrm{A}}(\mathbf{k},x)\,G^{\mathrm{R}}(\mathbf{k},-x)\,G^{\mathrm{A}}(\mathbf{k}+\mathbf{Q},x+\Omega)\,G^{\mathrm{R}}(\mathbf{k}+\mathbf{Q},-x-\Omega)\,, \qquad (38)
\end{aligned}
$$

where the integral region of $\mathbf{k}$ is restricted to one of the subbands. The final expression is to be calculated numerically. We use the Cuba library [7] to perform the numeric integral. Parameters and results are shown in the main text.

Supplementary References

[1] Buhmann, J. M., Ossadnik, M., Rice, T. M. & Sigrist, M. Numerical study of charge transport of overdoped $La_{2-x}Sr_xCuO_4$ within semiclassical Boltzmann transport theory. *Phys. Rev. B* **87**, 035129 (2013).

[2] Aslamasov, L. & Larkin, A. The influence of fluctuation pairing of electrons on the conductivity of normal metal. *Phys. Lett. A* **26**, 238–239 (1968).

[3] Larkin, A. & Varlamov, A. *Theory of Fluctuations in Superconductors* (Oxford Science Publications, Oxford, 2005).

[4] Leggett, A. J. Number-phase fluctuations in two-band superconductors. *Prog. Theor. Phys.* **36**, 901–930 (1966).

[5] Leggett, A. J. Theory of a superfluid Fermi liquid. II. collective oscillations. *Phys. Rev.* **147**, 119–130 (1966).

[6] Leggett, A. J. Theory of a superfluid Fermi liquid. I. general formalism and static properties. *Phys. Rev.* **140**, A1869–A1888 (1965).

[7] Hahn, T. Cuba—a library for multidimensional numerical integration. *Comput. Phys. Commun.* **168**, 78–95 (2005).
